# The Relationship between Proteinuria and Coronary Risk: A Systematic Review and Meta-Analysis

**DOI:** 10.1371/journal.pmed.0050207

**Published:** 2008-10-21

**Authors:** Vlado Perkovic, Christine Verdon, Toshiharu Ninomiya, Federica Barzi, Alan Cass, Anushka Patel, Meg Jardine, Martin Gallagher, Fiona Turnbull, John Chalmers, Jonathan Craig, Rachel Huxley

**Affiliations:** 1 The George Institute for International Health, Sydney, New South Wales, Australia; 2 University of Sydney, Sydney, New South Wales, Australia; Instituto Mario Negri, Italy

## Abstract

**Background:**

Markers of kidney dysfunction such as proteinuria or albuminuria have been reported to be associated with coronary heart disease, but the consistency and strength of any such relationship has not been clearly defined. This lack of clarity has led to great uncertainty as to how proteinuria should be treated in the assessment and management of cardiovascular risk. We therefore undertook a systematic review of published cohort studies aiming to provide a reliable estimate of the strength of association between proteinuria and coronary heart disease.

**Methods and Findings:**

A meta-analysis of cohort studies was conducted to obtain a summary estimate of the association between measures of proteinuria and coronary risk. MEDLINE and EMBASE were searched for studies reporting an age- or multivariate-adjusted estimate and standard error of the association between proteinuria and coronary heart disease. Studies were excluded if the majority of the study population had known glomerular disease or were the recipients of renal transplants. Two independent researchers extracted the estimates of association between proteinuria (total urinary protein >300 mg/d), microalbuminuria (urinary albumin 30–300 mg/d), macroalbuminuria (urinary albumin >300 mg/d), and risk of coronary disease from individual studies. These estimates were combined using a random-effects model. Sensitivity analyses were conducted to examine possible sources of heterogeneity in effect size. A total of 26 cohort studies were identified involving 169,949 individuals and 7,117 coronary events (27% fatal). The presence of proteinuria was associated with an approximate 50% increase in coronary risk (risk ratio 1.47, 95% confidence interval [CI] 1.23–1.74) after adjustment for known risk factors. For albuminuria, there was evidence of a dose–response relationship: individuals with microalbuminuria were at 50% greater risk of coronary heart disease (risk ratio 1.47, 95% CI 1.30–1.66) than those without; in those with macroalbuminuria the risk was more than doubled (risk ratio 2.17, 1.87–2.52). Sensitivity analysis indicated no important differences in prespecified subgroups.

**Conclusion:**

These data confirm a strong and continuous association between proteinuria and subsequent risk of coronary heart disease, and suggest that proteinuria should be incorporated into the assessment of an individual's cardiovascular risk.

## Abstract 2

Vlado Perkovic and colleagues show, through a systematic review and meta-analysis of cohort studies, that there is a strong and continuous association between proteinuria and subsequent risk of coronary heart disease.

## Editors' Summary

**Background.:**

Coronary heart disease (CHD) is the leading cause of death among adults in developed countries. With age, fatty deposits called atherosclerotic plaques coat the walls of arteries, the vessels that nourish the organs of the body by carrying blood and oxygen to them. Because they narrow the arteries, atherosclerotic plaques restrict the blood flow to the body's organs. If these plaques form in the arteries that feed the heart muscle (the coronary arteries), the result is CHD. The symptoms of CHD include shortness of breath and chest pains (angina). In addition, if a plaque breaks off the wall of a coronary artery, it can completely block that artery, which kills part of the heart muscle and causes a potentially fatal heart attack. Smoking, high blood pressure, high blood levels of cholesterol (a type of fat), having diabetes, being overweight, and being physically inactive are established risk factors for CHD. Treatments for CHD include lifestyle changes (for example, losing weight) and medications that lower blood pressure and blood cholesterol. The narrowed arteries can also be widened using a device called a stent or surgically bypassed.

**Why Was This Study Done?:**

In addition to the established risk factors for CHD, several other factors may also increase a person's risk of developing CHD, including kidney disease, which affects one in six adults to some degree. An early sign of kidney dysfunction is high amounts of a protein called albumin or of total proteins in the urine (albuminuria and proteinuria, respectively). Some studies have suggested that proteinuria is associated with an increased risk of CHD, but the results of these studies are inconsistent. Consequently, it is unclear whether proteinuria should be considered when assessing and managing an individual's CHD risk. In this study, the researchers undertake a systematic review (a study in which predefined search criteria are used to identify all the research on a specific topic) and a meta-analysis (a statistical method for combining the results of several studies) of published studies that have investigated the association between proteinuria and CHD.

**What Did the Researchers Do and Find?:**

The researchers' systematic review identified 26 published studies that provided estimates of the association between CHD risk and proteinuria and albuminuria by measuring baseline urinary protein and albumin levels in people who were then followed for several years to see whether they developed CHD. Nearly 170,000 individuals participated in these studies, which recorded more 7,000 fatal and nonfatal heart attacks and other coronary events. In the meta-analysis, proteinuria (urinary protein of more than 300 mg/d or dipstick 1+ or more) increased CHD risk by 50% after adjustment for other known CHD risk factors. Furthermore, individuals with microalbuminuria (a urinary albumin of 30–300 mg/d) were 50% more likely to develop CHD than those with normal amounts of urinary albumin; people with macroalbuminuria (urinary albumin of more than 300 mg/d) were more than twice as likely to develop CHD. Finally, the association between proteinuria and CHD did not differ substantially between specific subgroups of participants such as people with and without diabetes.

**What Do These Findings Mean?:**

These findings suggest that there is a strong, possibly dose-dependent association between proteinuria and the risk of CHD and that this association is independent of other known CHD risk factors, including diabetes. The finding that people with proteinuria have a 50% or greater increased risk of developing CHD than people without proteinuria may be a slight overestimate of the strength of the association between proteinuria because of publication bias. That is, studies that failed to show an association may not have been published. However, because this systematic review and meta-analysis includes several large population-based studies done in various parts of the world, these findings are likely to be generalizable. Thus, these findings support the inclusion of an evaluation of proteinuria in the assessment of CHD risk and suggest that medications and other strategies that reduce proteinuria might help to reduce the overall burden of CHD.

**Additional Information.:**

Please access these Web sites via the online version of this summary at http://dx.doi.org/10.1371/journal.pmed.0050207.

- The MedlinePlus encyclopedia has pages on coronary heart disease, atherosclerosis, and chronic kidney failure (in English and Spanish)
- Information is available from the US National Heart Lung and Blood Institute on coronary heart disease
- The UK National Health Service Direct health encyclopedia also provides information about coronary heart disease (in several languages)
- Information for patients and caregivers is provided by the American Heart Association on all aspects of heart disease.
- The British Heart Foundation also provides information on heart disease and on keeping the heart healthy

## Introduction

Over recent decades, substantial progress has been made in understanding the role that “classical risk factors”—namely blood pressure, smoking, cholesterol, diabetes, and obesity—play in the aetiology of coronary heart disease (CHD) [1–6]. Subsequent studies and meta-analyses have identified other factors that may provide additional important predictive information regarding the risk of CHD, including inflammatory markers [7], haemostatic factors [8,9], left ventricular hypertrophy, and markers of kidney dysfunction [10].

Kidney disease is highly prevalent worldwide [11–13], affecting approximately one in six adults in Western countries. An association between severe kidney failure and accelerated cardiovascular disease (CVD) has long been recognized [14]. Markers of early kidney disease, such as the presence of albumin or protein in the urine, have been reported to be associated with increased risk of CHD in the general population [15]. However, inconsistencies in both the direction and magnitude of the reported relationship have led to uncertainty about the practical benefit of measuring these indices in quantifying an individual's future coronary risk. Variations in the definition, measurement, and assessment of proteinuria have added to the confusion, with some studies reporting on the relationship between urinary albumin excretion (micro- or macroalbuminuria) and coronary risk, while others have used total urinary protein excretion, of which albumin is a component.

Previous reports have suggested a positive association between proteinuria and CHD risk [10], but the strength, consistency, and independence of the relationship have not been defined. Hence, the aim of the current study was to provide reliable estimates of the strength and nature of the association between proteinuria (urinary protein excretion of >300 mg/d), microalbuminuria (30–300 mg/d of urinary albumin excretion), and macroalbuminuria (>300 mg/d) with subsequent risk of CHD in the general population and in predefined subgroups (diabetes, gender, and ethnicity).

## Methods

### Data Sources and Searches

We performed a systematic review of the available literature according to the MOOSE guidelines [16] for the conduct of meta-analyses of observational studies. Relevant studies published between 1966 and November 2006 were identified from CINAHL (http://www.ebscohost.com/cinahl/), EMBASE (http://www.embase.com/), and MEDLINE (http://ovidsp.tx.ovid.com/spb/ovidweb.cgi and http://www.ncbi.nlm.nih.gov/sites/entrez?db=pubmed) using a combined text word and MeSH heading search strategy that included all spellings of proteinuria, albuminuria, microalbuminuria, or macroalbuminuria combined with coronary heart disease, ischemic heart disease, myocardial infarction, and angina pectoris, and limited to cohort studies. References from identified studies were manually scanned to identify any other relevant studies.

### Study Selection and Data Extraction

Cohort studies were included if they reported quantitative estimates of the age and sex-adjusted relative risk (RR) for fatal or nonfatal CHD associated with any level of albuminuria or proteinuria (Table 1), together with an estimate of variance (standard error or 95% confidence intervals [CIs]). Studies were excluded if the study population chiefly comprised pathological subgroups (including hospital-based populations or those that the majority of the study population comprised individuals with a history of myocardial infarction, chronic kidney disease, or renal transplantation), if they reported the estimate of effect with no means by which to derive the standard error, or if the estimate was not adjusted for age. Reports from clinical trials were excluded to avoid potential modification of the nature and magnitude of the relationship between proteinuria and CHD by the intervention studied (e.g., blood pressure lowering). Studies reported in languages other than English were included.

**Table 1:**
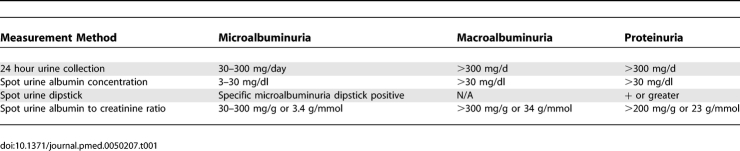
Definitions of Albuminuria and Proteinuria

CHD mortality was defined as death attributed to ICD9 (International Classification of Diseases and Related Health Problems, Ninth Revision) codes 410 to 414.9 and ICD10 codes I20.0 to I25.9. The prespecified definitions of proteinuria are shown in Table 1. The literature search and data extraction were conducted by two of the authors (CV and TN). Where there was disagreement over the eligibility of a study, three additional authors reviewed the paper until a consensus was reached (RH, VP, FB).

### Data Synthesis and Analyses

Summary estimates of RR were obtained using a random effects model. Separate summary estimates were obtained for the RR associated with microalbuminuria, macroalbuminuria, any albuminuria (micro or macro), and proteinuria. In addition, some studies reported sex-specific risk ratios for the strength of the association, and these were pooled if a single overall estimate was not provided.

The percentage of variability across studies attributable to heterogeneity beyond chance was estimated using the *I* ^2^ statistic [17]. The 95% CI of the *I* ^2^ statistic was calculated using the method described by Higgins et al. [18]. We investigated possible sources of heterogeneity by comparing summary results obtained when studies were grouped according to specific characteristics. Tests for heterogeneity were performed using meta-regression analysis. Publication bias was assessed using the Egger test and represented graphically using funnel plots plotting the natural log of the RR versus its standard error. The trim and fill analysis for publication bias was performed using Duval and Tweedie's methods [19]. A *p*-value below 0.05 was considered statistically significant in all analyses. All analyses were performed using STATA (Release 9.2; Stata Corporation, http://www.stata.com/).

## Results

### Literature Search and Characteristics of Studies

**Figure 1:**
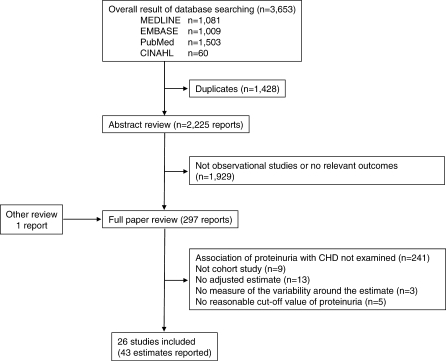
Identification Process for Eligible Studies

The electronic search yielded a total of 3,653 articles, of which 297 reports were reviewed in full text (see Figure 1). Of these, 26 studies were eligible for inclusion [20–45], including information on 169,949 individuals and 7,117 CHD events (27% fatal). Study size ranged from 146 to 90,363 participants and the average duration of participant follow-up was 4–27 y (Table 2).

**Table 2:**
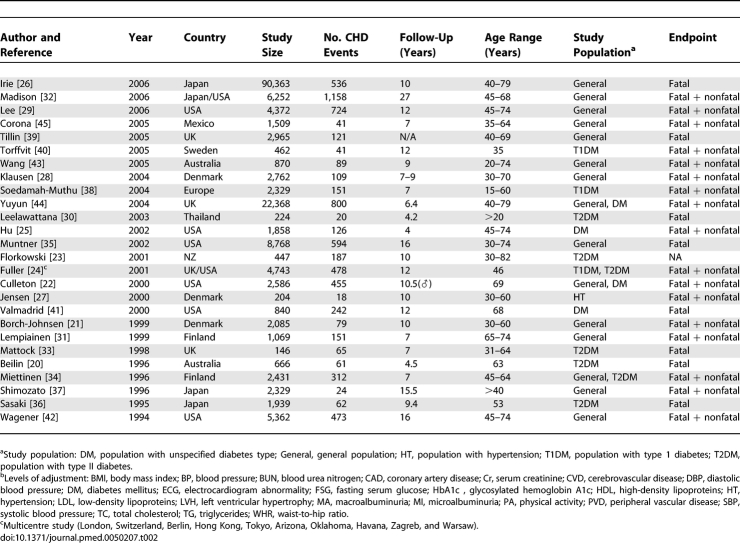
Characteristics of Studies Reporting on the Association between Proteinuria and Subsequent Risk of Coronary Heart Disease

**Table 2:**
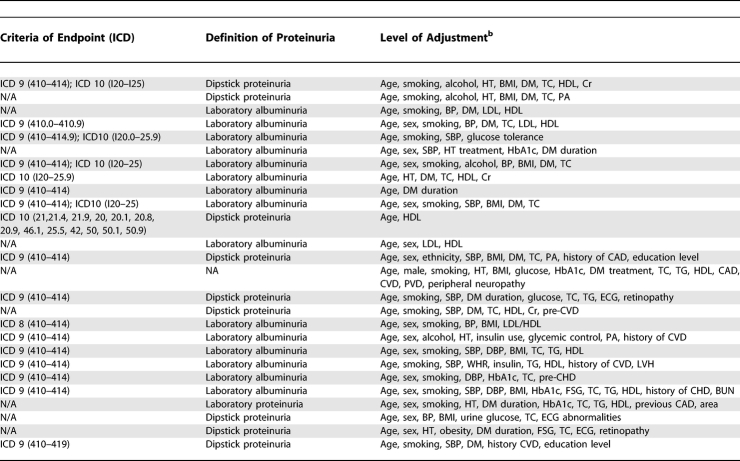
Extended.

In total, 26 studies reported the RR for CHD associated with a measure of proteinuria, and some reported the relationship for more than one threshold of proteinuria: ten studies reported on the association between total proteinuria and CHD; seven studies reported on the association between microalbuminuria and CHD; nine studies reported on the association between macroalbuminuria and CHD; and six studies reported on the association between any level of albuminuria and CHD. Levels of proteinuria were measured using either urinary dipstick tests for protein or the measurement of the UPCR (urinary protein–creatinine ratio) on spot urine specimens. The former was used in the majority of studies (*n* = 9). Levels of albuminuria were estimated by measurement of the UACR (urinary albumin–creatinine ratio) on spot urine specimens. Twenty-three studies reported hazard ratios, and three reported odds ratios. Twenty-five of the studies provided an estimate that had been adjusted for major CHD risk factors including blood pressure, smoking, diabetes, and cholesterol (Table 2).

### Association between Proteinuria and Subsequent Risk of CHD

**Figure 2:**
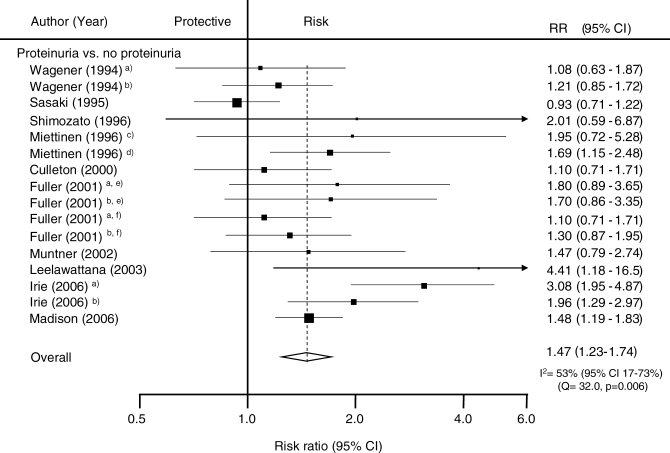
Summary Risk Ratio (95% Confidence Intervals) for the Association of Proteinuria with the Risk of Coronary Heart Disease in Population-Based Cohort Studies The black squares are inversely proportional to the variance of the study and the horizontal lines represent the 95% CIs. Footnotes: Four studies reported estimates separated according to subgroups including (a) female, (b) male, (c) people without diabetes, (d) people with diabetes, (e) people with type 1 diabetes, and (f) people with type 2 diabetes. References: Culleton 2000 [22]; Fuller 2001 [24]; Irie 2006 [26]; Leelawattana 2003 [30]; Madison 2006 [32]; Miettinen 1996 [34]; Muntner 2002 [35]; Sasaki 1995 [36]; Shimozato 1996 [37]; Wagener 1994 [42].

A total of 16 estimates from ten studies including 124,997 patients reported on the relationship between total proteinuria and the risk of subsequent CHD (Figure 2). The mean-weighted estimate from these studies indicated that individuals with proteinuria have an approximately 50% greater risk of CHD compared with those without the condition: RR 1.47 (95% CI 1.23–1.74). There was considerable heterogeneity across these studies (*p* < 0.006) but no significant evidence of publication bias (*p* = 0.11).

**Figure 3:**
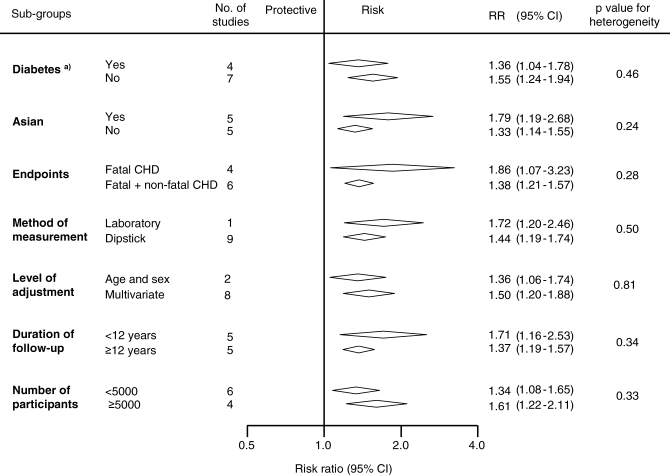
Examination of Potential Sources of Heterogeneity between Studies of Proteinuria and CHD According to Study or Participant Characteristics Conventions as in Figure 2. Footnote: (a) One study reported both risk estimates for individuals with and without diabetes.

Potential sources of heterogeneity in the strength of the association between proteinuria and CHD were examined by conducting sensitivity analyses (Figure 3). There was no evidence that the strength of the association differed according to diabetes status, ethnicity (Asian versus non-Asian, where most Asian participants were from Japan), fatal versus nonfatal CHD outcomes, laboratory versus dipstick measurement of proteinuria, duration of study follow-up, or study size (all *p*-values for heterogeneity > 0.1).

### Association between Albuminuria and Subsequent Risk of CHD

**Figure 4:**
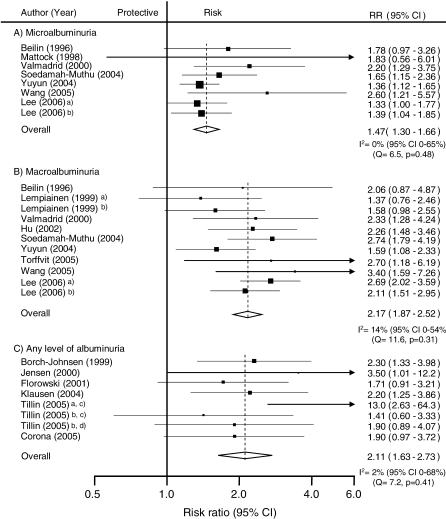
Summary Risk Ratio (95% Confidence Intervals) for the Association of Albuminuria with the Risk of Coronary Heart Disease in Population-Based Cohort Studies (A) Studies reporting risk estimate of microalbuminuria compared to normoalbuminuria. (B) Studies reporting risk estimate of macroalbuminuria compared to normoalbuminuria. (C) Studies reporting risk estimate of any level of albuminuria compared to normoalbuminuria. Conventions as in Figure 2. Footnotes: Three studies reported separated estimates according to subgroups including (a) female, (b) male, (c) non-Asian people, and (d) Asian people. References: Beilin 1996 [20]; Borch-Johnsen 1999 [21]; Corona 2005 [45]; Florkowski 2001 [23]; Hu 2002 [25]; Jensen 2000 [27]; Klausen 2004 [28]; Lee 2006 [29]; Lempiainen 1999 [31]; Mattock 1998 [33]; Soedarnah-Muthu 2004 [38]; Tillin 2005 [39]; Torffvit 2005 [40]; Valmadrid 2000 [41]; Wang 2005 [43]; Yuyun 2004 [44].

In the seven studies (*n* = 31,591 participants) for which information was available, individuals with microalbuminuria had a 50% greater risk of subsequent CHD compared with individuals without, and there was no evidence of heterogeneity among included studies: RR 1.47 (95% CI 1.30–1.66); *p* for heterogeneity = 0.48 (Figure 4). Evidence of significant publication bias was identified using the Egger test (*p* = 0.01), and after correction for its presence the estimate of the association was marginally reduced to 1.42 (95% CI 1.23–1.64). A total of nine studies (*n* = 34,834 participants) compared the risk of CHD among individuals with and without macroalbuminuria. Individuals in whom macroalbuminuria was detected had double the risk of CHD compared with individuals without, with no evidence of publication bias: RR 2.17, 95% CI 1.87–2.52 (Figure 4). Even after accounting for the fact that we are comparing different levels of albuminuria with the same control group by using the Bonferroni correction, the pooled estimates of microalbuminuria and macroalbuminuria remained highly significant (both *p* < 0.0001 after Bonferroni correction).

In those six studies (*n* = 9,972 participants) that reported on the association between any level of albuminuria the RR for CHD among individuals with albuminuria was twice that of normoalbuminuric individuals with no evidence of publication bias: RR 2.11, 95% CI 1.63–2.73; *p* for heterogeneity = 0.41 (Figure 4).

**Figure 5:**
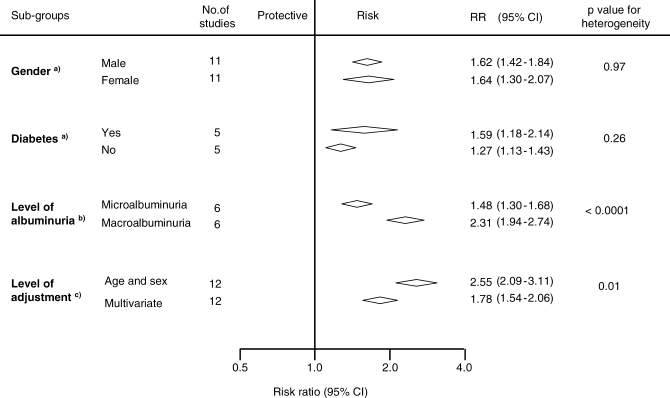
Subgroup Analysis of Comparisons within Studies Reporting Associations of Proteinuria and Albuminuria with Coronary Heart Disease Risk Conventions as in Figure 2. Studies reporting both risk estimates for individuals with and without risk factor were included for the analysis. Footnotes: (a) Studies reporting both risk estimates for individuals with and without diabates were included for the analysis. (b) Studies reporting both risk estimates for individuals with microalbuminuria and those with macroalbuminuria were included for the analysis. (c) Studies reporting both age-adjusted estimates and multivariate-adjusted estimates were included for the analysis.

To examine whether there was a dose–response association between albuminuria and CHD the following analysis was restricted to those six studies (*n* = 31,445 participants) that had reported separately on the association between microalbuminuria and macroalbuminuria and subsequent CHD (Figure 5). Among these studies, compared with normoalbuminuric individuals, those with microalbuminuria had a 50% greater risk of CHD (RR 1.48, 95% CI 1.30–1.68) and in those with macroalbuminuria, the risk of CHD was more than doubled (RR 2.55, 95% CI 2.09–3.11; *p* for heterogeneity < 0.0001).

### Impact of Adjustment for Major Cardiovascular Risk Factors on the Strength of the Association

A total of 12 studies (*n* = 138,003 participants) reported estimates of the strength of the association between either proteinuria or albuminuria that were adjusted first, by age and then by other known cardiovascular risk factors (i.e., blood pressure, smoking, lipids and diabetes). The overall age-adjusted summary estimate was 2.55 (95% CI 2.09–3.11), which was reduced by approximately 50% to 1.78 (95% CI 1.54–2.06; *p* for heterogeneity = 0.01, Figure 5).

## Discussion

This overview of the epidemiological evidence suggests that proteinuria is independently associated with increased risk of subsequent CHD. The results from this meta-analysis of 26 cohort studies, including information on over 7,000 CHD events among almost 170,000 individuals, suggest that people with proteinuria have a risk of CHD that is at least 50% greater than those without. Moreover, there was some evidence to indicate a dose–response relationship such that the strength of the association was substantially higher among individuals with macroalbuminuria compared with those with microalbuminuria. Furthermore, the relationship was consistent across diverse population subgroups including individuals with and without diabetes. The included studies were largely population-based suggesting that these findings are broadly generalisable and less likely to have been affected by interventions (e.g., blood pressure lowering) than data from clinical trials.

The magnitude of the risk is similar to that associated with many classical risk factors, and stronger than many more recently discovered risk factors [7,9] suggesting that the addition of proteinuria may improve the predictive ability of commonly used cardiovascular risk prediction formulae. This is being increasingly recognized, with the most recent cardiovascular disease prevention guidelines from the American Heart Association [46] suggesting that individuals with proteinuria should be considered to be at similar risk to people with established CHD. Our data suggest that proteinuria is likely to improve the ability to predict coronary risk. Further studies will be required to assess whether the performance of commonly used risk prediction tools can be improved by the inclusion of proteinuria in cardiovascular risk prediction formulae.

Although these data suggest that albuminuria may be a stronger predictor of coronary risk than urinary total protein excretion, this may be an artefact of the data, since the majority of studies that measured urinary protein used dipstick tests, which have a lower sensitivity and specificity than do laboratory estimates. Unfortunately, as only one of the included studies used laboratory estimates to measure proteinuria, we had limited statistical power in the sensitivity analysis to be able to detect any real difference between the two methods. The included studies did not use a specific methodology for the collection of the urine specimens, adding a further potential source of variability [47].

The relationship between proteinuria and CHD was similar in individuals with and without diabetes, and in the other subgroups studied. We were unable to assess whether proteinuria conferred an increased risk of coronary disease separately to other manifestations of chronic kidney disease, although others have recently reported that these risks are additive [48]. Although the impact of therapies that reduce proteinuria (e.g., blood pressure–lowering agents) have been the subject of reasonably large trials in people with diabetes and proteinuria [49,50], fewer data are available regarding the effects of these therapies in individuals without diabetes and with proteinuria. The Heart Outcomes Protection Evaluation (HOPE [51]) suggested that a somewhat greater magnitude of cardiovascular protection may be associated with the use of the angiotensin-converting enzyme ramipril in participants with albuminuria at baseline (relative risk reduction of 26% versus 15%), although the statistical significance of this difference was not reported. Data from other trials suggest that the reduction in urinary protein excretion achieved in an individual predicts the reduction in the risk of subsequent cardiovascular events [52,53]. The confirmation of a differential cardiovascular protective efficacy for blood pressure–lowering agents according to baseline proteinuria in future studies would further increase the value of adding proteinuria to cardiovascular risk prediction tools that guide decisions regarding the use of preventative therapies.

Our systematic review could not assess whether proteinuria plays a causative role in the development of CHD, as this would require evidence from randomised controlled trials of therapies that acted mainly by reducing proteinuria. It has been suggested that proteinuria may simply be a marker of early vascular disease, as propounded in the “Steno hypothesis.” This hypothesis contends that the presence of proteinuria reflects widespread early vascular disease and abnormal endothelial function (including that in the glomerular vasculature), and as such may be a marker of the severity and duration of other risk factors (e.g., elevated blood pressure) rather than playing a pathogenic role per se [54].

An important limitation of the current analyses is its reliance upon published summary data (rather than individual participant data), which impairs the examination of the impact of adjustment for known CHD risk factors. In those studies that provided both adjusted and unadjusted estimates, the association between proteinuria and CHD was significantly attenuated by about 50% after adjustment. Such a significant attenuation in effect size suggests that residual confounding is likely to remain and, further, that the summary result presented here may be an overestimate of the true magnitude of the association between proteinuria and CHD. Some evidence of publication bias was also identified, although after correcting for it, the associations remained strong and significant. Conversely, the strength of the relationship may have been underestimated due to the impact of regression dilution bias [55], in which a single measurement of a risk factor may underestimate the strength of its relationship to a disease. The effect of regression dilution bias is supported by the findings of the study by Madison et al. [32], in which proteinuria present on two separate measurements was associated with a much higher risk of subsequent coronary heart disease than transient proteinuria (RR 3.72, 95% CI 2.62–5.27 versus 1.48, 1.19–1.83). Despite these limitations, the results of this systematic review represent the most precise and accurate estimate of the strength of the relationship between proteinuria and CHD currently available.

The findings of this study therefore strongly support a role for the evaluation of proteinuria in the prediction of CHD risk. They suggest that the use of strategies to reduce proteinuria, and better targeting of other cardioprotective therapies, may help to reduce the overall burden of CHD. Studies to assess the impact of these strategies are warranted.

## Abbreviations

CHD: coronary heart disease
CI: confidence interval
CVD: cardiovascular disease
RR: relative risk
